# Pathogenesis of Distal Symmetrical Polyneuropathy in Diabetes

**DOI:** 10.3390/life12071074

**Published:** 2022-07-19

**Authors:** Sasha Smith, Pasha Normahani, Tristan Lane, David Hohenschurz-Schmidt, Nick Oliver, Alun Huw Davies

**Affiliations:** 1 Section of Vascular Surgery, Department of Surgery and Cancer, Imperial College London, London W6 8RF, UK; sasha.smith@imperial.ac.uk (S.S.); p.normahani@imperial.ac.uk (P.N.); tristan.lane@imperial.ac.uk (T.L.); 2 Imperial Vascular Unit, Imperial College Healthcare NHS Trust, London W6 8RF, UK; 3 Department of Vascular Surgery, Cambridge University Hospitals NHS Foundation Trust, Cambridge CB2 0QQ, UK; 4 Pain Research Group, Department of Surgery and Cancer, Imperial College London, London SW10 9NH, UK; d.hohenschurz-schmidt19@imperial.ac.uk; 5 Section of Metabolic Medicine, Department of Metabolism, Digestion and Reproduction, Imperial College London, London W2 1PG, UK; nick.oliver@imperial.ac.uk; 6 Division of Medicine and Integrated Care, Imperial College Healthcare NHS Trust, London W2 1NY, UK

**Keywords:** diabetes, diabetic neuropathy, distal symmetrical polyneuropathy, neuropathic pain, pathogenesis, hyperglycemia, central nervous system, glycemic control, pain management, spinal cord stimulation

## Abstract

Distal symmetrical polyneuropathy (DSPN) is a serious complication of diabetes associated with significant disability and mortality. Although more than 50% of people with diabetes develop DSPN, its pathogenesis is still relatively unknown. This lack of understanding has limited the development of novel disease-modifying therapies and left the reasons for failed therapies uncertain, which is critical given that current management strategies often fail to achieve long-term efficacy. In this article, the pathogenesis of DSPN is reviewed, covering pathogenic changes in the peripheral nervous system, microvasculature and central nervous system (CNS). Furthermore, the successes and limitations of current therapies are discussed, and potential therapeutic targets are proposed. Recent findings on its pathogenesis have called the definition of DSPN into question and transformed the disease model, paving the way for new research prospects.

## 1. Introduction

Distal symmetrical polyneuropathy (DSPN) is a serious complication of diabetes with a lifetime prevalence of more than 50% in people with diabetes [1], a population globally estimated at half a billion and expected to rise to 700 million by 2045 [2]. DSPN, also still commonly referred to as “diabetic peripheral neuropathy”, is defined by the American Diabetes Association as “the presence of symptoms and/or signs of peripheral nerve dysfunction in people with diabetes after the exclusion of other causes” [3] (p. 138). It is the most prevalent and studied clinical presentation of diabetic neuropathy, accounting for 75% of all cases [4], with the strongest evidence for its pathogenesis, and is thus the focus of this review article. Other clinical presentations of diabetic neuropathy, such as autonomic neuropathies, acute painful-distal sensory polyneuropathies (hyperglycemia- or treatment-induced), focal or multifocal neuropathies, mononeuropathy, mononeuropathy multiplex, radiculoplexus neuropathy and entrapment neuropathies [5], are beyond the scope of this article; however, we refer the reader to relevant review articles [4,6,7,8,9,10,11,12,13,14,15,16,17,18,19,20]. Also, the pathogeneses of other linked diabetes-related complications, such as retinopathy, foot ulceration and Charcot neuropathic osteoarthropathy, are not discussed in this article.

DSPN is known as a “length-dependent” neuropathy because it is thought to begin in the distal nerve endings of the longest neurons in the limbs and spread proximally from its “stocking and glove” pattern. Symptoms include weakness, unsteadiness, chronic pain and/or numbness in the hands and feet, which can result in a reduced quality of life as well as an increased risk of falls and sleep disturbances [21,22,23]. Furthermore, DSPN is an important risk factor for diabetic foot ulceration, which has a 5-year rate of lower limb amputation and mortality of 10% and 40%, respectively [24]. Diabetic foot ulcers and amputations cost the National Health Service (NHS) in the United Kingdom (UK) approximately GBP 1 billion per year [24,25], while Medicare and private insurers in the United States (USA) spend between USD 9 billion and USD 13 billion per year [26]. The financial burden of diabetic foot complications remains high globally; although annual figures are not available, a recent systematic review of 55 studies reported that the cost per amputation in developed countries ranges between Int$ 35,000 and Int$ 45,000 (international dollars) [27].

Although more than half of people with diabetes develop DSPN [1], its pathogenesis is still relatively unknown. This has limited the development of novel disease-modifying therapies because there is uncertainty around potential therapeutic targets and as to why previous promising therapies have failed in clinical trials. For example, aldose reductase inhibitors (ARIs) have the potential to prevent overactivity of the polyol pathway, a key pathogenic mechanism in DSPN; however, a previous Cochrane systematic review did not identify a single randomized controlled trial (RCT) demonstrating significant differences in ARIs versus placebo in the treatment of DSPN, and it is unclear why treatment efficacy could not be achieved [28]. As a result, the industry has been hesitant to invest in such research, despite the fact that it is critical for further knowledge and given that current management strategies focus on prevention and pain management, which often fail to achieve long-term efficacy [29].

Considering the strong link between diabetes and nerve dysfunction (diabetes is the top comorbidity of peripheral neuropathy globally [30]), it was previously assumed that diabetes developed secondary to nerve damage. Although intriguing, this has been refuted, and basic scientists have primarily focused on the effects of hyperglycemia on peripheral neurons [19]. A seminal article by Brownlee [31] proposed a unifying mechanism for all diabetic complications, hypothesizing that long-term hyperglycemia causes the mitochondrial electron transport chain to overproduce superoxide, resulting in an increase of reactive oxygen species (ROS) and several downstream pathogenic mechanisms. It was suggested that neurons and microvessels in particular were susceptible to hyperglycemia due to their shared inability to balance intracellular glucose levels, resulting in diabetic neuropathy. The discovery of this upstream mechanism provided new therapeutic targets and indicated that the pathogenic mechanisms in neurons and microvessels may be similar [31].

In the same year, Duby et al. [18] conducted an intensive review of diabetic neuropathy, which they classified as a disease affecting the peripheral nervous system. The role of hyperglycemia in its pathogenesis was also acknowledged, as well as the commonalities between neuron and microvessel pathology. The significance of microvasculature changes was stressed, highlighting that diabetic neuropathy is “neurovascular” and a “microvascular complication”. The authors emphasized the strong interconnection and co-dependency between the nervous and the vascular tissues but cautioned that they may respond differently to treatment [18]. Since that review, an increasingly integrated view has been adopted, acknowledging the interplay of other metabolic factors, such as hyperlipidemia and impaired insulin signaling. Feldman et al. [32] recently provided an overview of these mechanisms and their interactions, suggesting how their contributions to DSPN may vary by cell type, disease type and duration, emphasizing the need for personalized treatments that wholly target nerve metabolism [32].

More recently, there has been a neuroimaging focus where advanced techniques have been utilized to demonstrate that early nerve damage may involve both the peripheral and the central nervous systems, drawing the definition of DSPN into question. Selvarajah et al. [33] presented evidence for central nervous system (CNS) involvement in DSPN, including potential differences between people with painful and painless phenotypes. These developments have transformed the DSPN disease model, paving the way for new research prospects [33]. Despite these advances in disease pathogenesis, there are currently no successful disease-modifying therapies for DSPN that target all diabetes subtypes.

A current review of DSPN pathogenesis is vital for directing the development of novel therapies, providing additional insight into why some therapies have failed and bridging the gap between available therapies and the mechanisms they may target. The aim of this article is to provide a narrative review of DSPN pathogenesis, covering pathogenic changes in the peripheral nervous system, microvasculature and CNS. Furthermore, the successes and limitations of current therapies are discussed, and potential therapeutic targets are proposed.

## 2. Neuropathology

DSPN is typically characterized by dysfunction in myelinated and unmyelinated peripheral neurons. Painful DSPN is associated with dysfunction predominantly in small Aδ (thinly myelinated) and C (unmyelinated) fibers, whereas painless or insensate DSPN is associated with predominantly large A fiber (myelinated) dysfunction, though mixed small and large fiber loss is common [3]. Microvascular disease can occur in both painful and painless DSPN, but in particular, dysfunction in microvascular blood flow is associated with pain [34]. The neuropathological features of DSPN have been studied most extensively in sensory neurons of animal models and cell cultures, but key studies on human nerve samples from people with DSPN following a biopsy have also been conducted.

**Figure 1:**
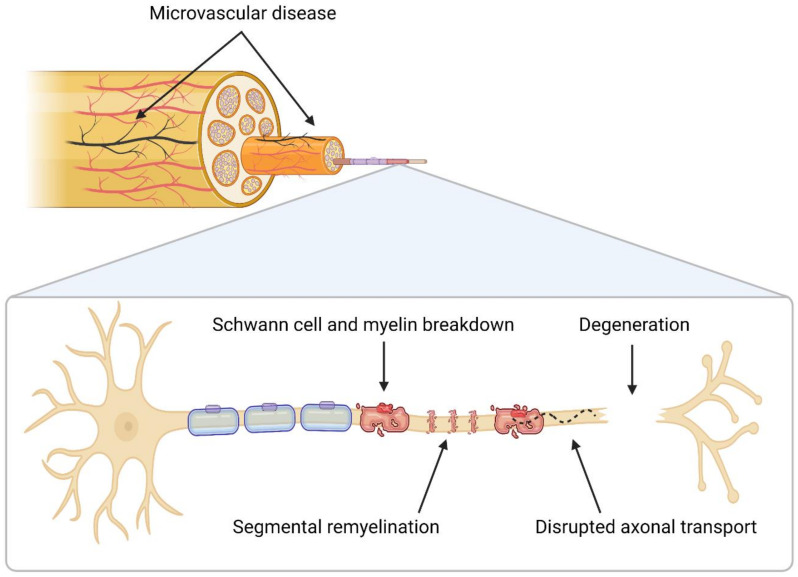
**Neuropathological findings of distal symmetrical polyneuropathy in diabetes.** Early breakdown of the myelin sheath and Schwann cells occurs. Schwann cells dissociate from axons even in unmyelinated neurons. Axonal transport and signaling are disrupted, potentially also at the axo–glial interface, resulting in decreased neurotrophic factors and, eventually, distal axonal loss that progresses length-dependently.

In myelinated neurons (Figure 1), the myelin sheath is disrupted early on in the development of DSPN. A study of sural nerve samples biopsied from 12 people with mild DSPN at baseline and 9 years revealed internodal changes, segmental demyelination and remyelination but no axonal degeneration at follow-up compared to match controls, indicating that axons are targeted later in disease progression [35]. Unmyelinated fibers from the same nerve samples showed a high number of disassociated Schwann cells and axonal sprouts when compared to matched controls, indicating concurrent degeneration and regeneration [35]. Evidence suggests that these changes, as well as those of small thinly myelinated (Aδ) fibers, occur before changes in large myelinated (A) fibers, though they are difficult to detect due to the regenerative nature of unmyelinated neurons [35,36]. This prominent study conducted by Malik et al. [35] provided important insights into the pathogenesis of DSPN by enrolling participants with minimal rather than advanced disease. In addition, the findings sparked interest in the function of Schwann cells in DSPN, but there have been few further studies investigating this in patients. Instead, there have been studies on Schwann cell cultures and animal models, which have shown that in a hyperglycemic state, Schwann cells produce less neurotrophic factors and undergo apoptosis [37,38,39]. It has been proposed that these changes may independently contribute to axonal loss, and thus Schwann cells may be considered therapeutic targets [32,35,40].

Disrupted axonal transport and signaling is another important neuropathological finding [41]. A comparison of sural nerve samples from people with diabetes and control subjects has shown significant differences in cytoskeletal proteins [42]. Furthermore, expression and transport of neurotrophic factors are significantly reduced at 6 weeks in streptozotocin-diabetic rats, a common animal model for diabetes, compared to control rats [43]. Transport may also be disrupted at the axo–glial interface, which may initiate or perpetuate axonal loss. For example, in BB Wistar rats, another animal model for diabetes, there was significant loss of axo–glial junctions that could not be reformed with glycemic control [44].

Early distal axonal loss occurs in both myelinated and unmyelinated neurons and appears to evolve in a length-dependent manner, affecting the distal nerve endings of the longest neurons in the lower limbs first and “dying back” proximally towards the perikarya [32,36,45]. However, gradual changes at the perikarya in the dorsal root ganglia have also been observed during early DSPN in both streptozotocin and BB Wistar rats [46,47], suggesting that dysfunction may occur here first and then signal distal axonal loss [48]. More recent neuroimaging data from patients, on the other hand, suggest that DSPN may begin with damage to the spinal cord (discussed later, see ‘*Section 4.1*. *Spinal Cord*’) [49].

The closely connected nerve microvasculature is also dysfunctional. Studies in people with diabetic neuropathy following biopsy or photography have shown changes in basement membrane density, pericyte function, endothelial cell growth and the formation of arteriovenous shunts, all signifying ischemic damage [50,51,52,53]. This has also been shown to reduce angiogenic factors in patients, such as vascular endothelial growth factor, which are neuroprotective [54]. Microvascular disease has been recognized as a distinct driver of DSPN, and its severity correlates with patient outcomes, such as nerve conductivity [50,53,55,56,57].

## 3. Metabolic Mechanisms

**Figure 2:**
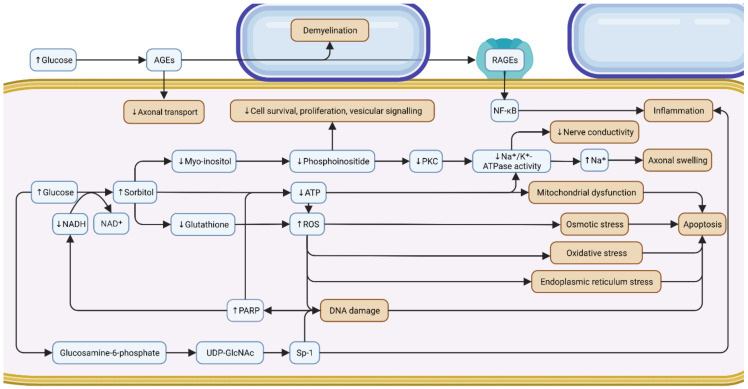
**Metabolic mechanisms of hyperglycemia in distal symmetrical polyneuropathy in diabetes.** This cross-sectional diagram of a peripheral myelinated sensory neuron depicts the various hypothesized metabolic mechanisms of hyperglycemia (polyol, glycation, protein kinase C, poly (ADP-ribose) polymerase and hexosamine pathways) and their interconnection, which are critical in DSPN pathogenesis and neuronal dysfunction. As shown, only the glycation pathway via AGEs acts on an extracellular level, while the others act on an intracellular level. Blue labels represent molecules and red labels represent neuropathic outcomes. ↑ refers to an increase and ↓ refers to a decrease. AGEs, advanced glycation end products; ADP, adenosine diphosphate; ATP, adenosine triphosphate; NA^+^, sodium ion; NAD^+^, nicotinamide adenine dinucleotide; NADH, nicotinamide adenine dinucleotide plus hydrogen; Na^+^/K^+^-ATPase, sodium–potassium ATPase; NF-ĸB, nuclear factor kappa B; PARP, poly (ADP-ribose) polymerase; PKC, protein kinase C, RAGEs, receptors for advanced glycation end products; ROS, reactive oxygen species; Sp1, specificity protein 1; UDP-GlcNAc, uridine diphosphate N-acetylglucosamine.

The primary drivers of DSPN pathogenesis are hyperglycemia, hyperlipidemia and impaired insulin signaling, which result in a variety of downstream pathogenic metabolic mechanisms. The most studied pathogenic environment, hyperglycemia, causes the overaction of the polyol, glycation, protein kinase C (PKC), poly (ADP-ribose) polymerase (PARP) and hexosamine pathways (Figure 2). All of these mechanisms lead to oxidative stress and cause concurrent nerve and microvascular damage.

### 3.1. Hyperglycemia

Hyperglycemia activates the following pathways in DSPN.

#### 3.1.1. Polyol

The aldose reductase reaction, in which the enzyme aldose reductase reduces glucose to produce sugar alcohols, primarily sorbitol, is the first stage in the polyol pathway. This reaction necessitates the conversion of nicotinamide adenine dinucleotide plus hydrogen (NADH) to nicotinamide adenine dinucleotide (NAD^+^). Usually, NADH levels are maintained because NAD^+^ is transformed back to NADH by the enzyme sorbitol dehydrogenase [58]. However, in people with diabetes and poor glycemic control, there is overactivation of the pathway, resulting in increased sorbitol and decreased NADH [31]. This is associated with decreased myo-inositol, a sugar derivative required for peripheral nerve function and development [59]. When myo-inositol levels are low, less phosphoinositide is converted, a molecule linked to cell survival, proliferation, vesicular signaling and glucose metabolism [59,60,61]. This subsequently reduces protein kinase C (PKC), which affects sodium–potassium ATPase (Na^+^/K^+^-ATPase), an enzyme strongly linked to nerve conductivity [61,62,63,64,65]. An accumulation of Na^+^ due to Na^+^/K^+^-ATPase dysfunction leads to axonal swelling [66,67]. In addition, increased sorbitol and reduced NADH levels lead to reduced glutathione, increased levels of ROS and increased cellular osmolarity. As a result, osmotic, endoplasmic reticulum and oxidative stress ensue, which, combined with a decrease in adenosine triphosphate (ATP) production causes mitochondrial damage, DNA damage and a decrease in nerve blood supply, accelerating apoptosis [68]. Mitogen-activated protein kinase (MAPK), an enzyme implicated in both protective and apoptotic pathways, is linked to aldose reductase and oxidative stress and may also contribute to DSPN pathogenesis [69].

#### 3.1.2. Glycation

A non-enzymatic reduction of glucose is glycation, or the attachment of sugars to proteins or lipids, which leads to the production of advanced glycation end products (AGEs) [70]. Other sugars, such as glucose-6-phosphate and fructose, also undergo glycation in the cell, and at a greater rate than glucose [71]. Although AGEs can form on a variety of cells in the heart, lungs, kidneys and liver, for example, their significance in DSPN is due to their production on axons (both myelinated and unmyelinated), pericytes, endothelial and Schwann cells [71,72,73]. AGEs also interfere with neurofilaments and microtubules in nerves, which may impede axonal transport, and their formation on myelin may result in localized demyelination [72,74]. AGEs on microvessels limit neuronal blood flow by increasing vascular permeability, inhibiting vasodilation via nitric oxide (NO) interference, inducing cytokine release and increasing oxidative stress [72,75,76,77,78]. AGEs also interact with receptors for advanced glycation end products (RAGEs), which signal to nuclear factor kappa B (NF-ĸB), a protein that controls several inflammatory markers, resulting in neurodegeneration and the inability to auto-repair [76]. The inconsistent approaches employed across studies to detect AGEs on cells, however, impact the quality of research on the glycation pathway [79].

#### 3.1.3. Protein Kinase C (PKC)

The exact role of PKC and its activator diacylglycerol (DAG) in DSPN is uncertain. Overactivation of the polyol pathway, as earlier mentioned, reduces myo-inositol, which lowers DAG and hence PKC, affecting Na^+^/K^+^-ATPase and leading to axonal swelling and reduced nerve conductivity [61,62,63,64,65,66,67]. Other studies have shown that hyperglycemia stimulates PKC, particularly in microvessels, and is associated with vascular modifications such as changes in endothelium and extracellular matrix structure, vasoconstriction, cell proliferation, angiogenesis, cytokine initiation and leukocyte linkages [80]. This is most likely explained by cell differences in hyperglycemia pathways and warrants further investigation [81]. Interestingly, AGEs stimulate PKC in mesangial and skeletal muscle cells, but this connection has yet to be investigated in DSPN [82,83,84].

#### 3.1.4. Poly (ADP-Ribose) Polymerase (PARP)

DSPN results in an influx of free radicals, oxidants, ROS and reactive nitrogen species in neurons. When, for example, NO and superoxide combine, peroxynitrite is generated, which causes DNA strand breaks and activates PARP, a DNA repair enzyme [85,86]. Overactivation of PARP depletes energy resources, as demonstrated by lower NAD^+^ and ATP levels, and results in reduced nerve conductivity [87]. This further enhances oxidative and nitrosative stress, mitochondrial damage, as well as apoptosis [88]. PARP also appears to be overexpressed in microvessels and may cause a similar energy-depleting process [87]. PARP may have the potential to inflict further damage by activating the MAPK, glycation and PKC pathways, as well as by influencing transcription factors and gene expression [87,89].

#### 3.1.5. Hexosamine

Hyperglycemia raises fructose-6-phosphate levels, causing the hexosamine pathway to flux. Fructose-6-phosphate and glutamine react to form glucosamine-6-phosphate [90], which can directly induce oxidative stress and lead to mitochondrial damage and apoptosis [31,91]. Eventually, uridine diphosphate N-acetylglucosamine (UDP-GlcNAc) is formed, which normally serves as a substrate for proteoglycan synthesis and the O-linked glycosylation of certain proteins, however, in DSPN is partly misdirected to transcription factors such as Sp-1 [92]. Sp-1 regulates the levels of insulin and lipids and the expression of transforming growth factor β1 and plasminogen activator inhibitor-1 and has a role in inflammation, DNA damage and apoptosis [92,93,94]. Recent evidence suggests that PKC may mediate this pathway [95].

### 3.2. Hyperlipidemia

Hyperlipidemia is a risk factor particularly for people with type 2 diabetes (T2DM). Excess free fatty acids (FFA) are broken down by β-oxidation, which produces ROS and inflammatory markers, whilst activating macrophages. Acetyl-CoA levels rise because of β-oxidation (and glucose metabolism), and acetyl-CoA is transformed to acylcarnitines, which in excess are toxic to neurons and Schwann cells and induce ATP depletion, mitochondrial dysfunction, oxidative stress and phagocytosis [32,96]. Low-density lipoproteins (LDLs) are oxidized by ROS and attached to oxidized LDL receptor 1 (LOX1), Toll-like receptor 4 (TLR4) and RAGEs. This further stimulates inflammatory markers, such as caspase 3, and increases superoxide, resulting in DNA damage and apoptosis. Oxidation of cholesterol further induces apoptosis via oxysterols [32].

### 3.3. Impaired Insulin Signaling

It has been proposed that insulin activates neuronal survival mechanisms by stimulating the production of neurotrophic and neuroprotective factors, and C-peptide directly repairs structural and functional axonal defects. In people with type 1 diabetes (T1DM), as levels of insulin and C-peptide fall, Na^+^/K^+^-ATPase and NO are disrupted, resulting in neuronal dysfunction, axonal swelling, oxidative stress and apoptosis. Although stable insulin levels can be achieved with careful treatment, serum levels do not always correspond to neuronal levels [48,97]. In people with T2DM, C-peptide aggregates and possibly links with macrophages and monocytes [98], which may be a defense mechanism that turns inflammatory if activated in excess [48]. Insulin resistance decreases Akt, which results in mitochondrial dysfunction, oxidative stress and apoptosis [99].

## 4. Central Nervous System (CNS) Changes

The link between diabetes and changes in the central nervous system (CNS) is well established. Diabetes is associated with a two-fold increased risk of stroke and depression [100,101], as well as 1.5-fold increased risk of dementia [102]. Neuroimaging studies have revealed that people with DSPN may have additional structural and functional changes in the CNS and demonstrated differences between people with painless and painful DSPN. However, these observations have been frequently made in pilot studies with small sample sizes or based in samples only including males with T1DM [33]. Nonetheless, the preliminary evidence is encouraging, and future research should focus on identifying the interconnected mechanisms between the peripheral and the central nervous systems in DSPN, as these may be therapeutic targets.

### 4.1. Spinal Cord

DSPN is linked to changes at the spinal cord. A pilot study enrolled 19 people with DSPN, 10 diabetics without DSPN and 10 healthy controls. Magnetic resonance imaging (MRI) revealed that the DSPN group had a significantly reduced mean spinal cord cross-sectional area at C4/5 and T3/4 compared to the other groups [103]. A subsequent, larger study (n = 113) validated these findings and also demonstrated that people with sub-clinical DSPN had significantly reduced mean spinal cord cross-section areas compared to people with no DSPN [49]. This suggests that spinal cord degeneration may occur at the same time as peripheral nerve degeneration, if not earlier, and spinal cord cross-sectional area could be a potential biomarker [49]. While early involvement of the spinal cord contradicts the “dying back” theory of DSPN [33], it may be a CNS form of length-dependent progression [49]. As the latter study only included males with T1DM, it is unclear if there are any differences depending on sex and in people with T2DM.

### 4.2. Brainstem

A pilot study using diffuse tensor MRI assessed connections between the brainstem and the somatosensory cortex. It was found that patients with DSPN (n = 6) had fewer connections compared to healthy volunteers (n = 4); however, the difference was not statistically significant [104]. If the findings were significant in an adequately powered trial, they may suggest that DSPN does not evolve in a length-dependent manner since it also affects second-order neurons [33].

Another pilot study using fMRI found that patients with painful DSPN (n = 14) have altered ventrolateral periaqueductal gray function following heat stimulation compared to people with painless DSPN (n = 12), and this was linked to their sporadic and allodynic pain. These results suggest that disruption of the periaqueductal gray, a mediator in descending pain pathways, may be a pathogenic mechanism contributing to painful DSPN [105].

### 4.3. Thalamus

Proton magnetic resonance spectroscopy has been used to assess neuro-molecular changes in the thalamus of people with DSPN. A pilot study demonstrated that people with DSPN (n = 10) had lower N-acetylaspartic acid (NAA)/creatine and NAA/choline ratios than diabetics without DSPN (n = 8) and healthy controls (n = 6). These differences were captured utilizing long echo time, indicating thalamic dysfunction rather than neuronal loss, as short echo time did not differ significantly [106]. Similarly, because the study only included males with T1DM, its generalizability is limited.

A pilot thalamic microvascular perfusion study has also demonstrated differences in the thalamus between people with painful and painless DSPN. Patients with painful DSPN (n = 5) had significantly higher thalamic relative cerebral blood volume, a hemodynamic measure for cerebral perfusion, than those with painless DSPN (n = 7) and diabetics with no DSPN (n = 6) [107]. This suggests thalamic neurons may act as pain generators in diabetes, possibly through thalamic vascularity; however, large-scale studies are needed to explore this theory further [33].

### 4.4. Somatosensory Cortex

CNS changes in DSPN have been demonstrated most widely in the somatosensory cortex. A study of 54 people (painful DSPN n = 9, painless DSPN n = 9, diabetics without DSPN n = 18, healthy controls n = 18) following volumetric brain MRI revealed that gray matter volume in regions involved in somatosensory processing are significantly reduced in people with DSPN (both painful and painless) compared to people without DSPN (diabetic and healthy controls). Significant differences were also observed at the supramarginal gyrus of the somatosensory association cortex and the cingulate cortex. These changes may simply be a consequence of decreased activity in peripheral neurons. Longitudinal studies are therefore needed to determine when this damage occurs [108].

The somatosensory cortex has also been studied with proton magnetic resonance spectroscopy. People with DSPN (n = 50) had lower NAA captured via short echo term sequences compared to diabetics without DSPN (n = 20) and healthy controls (n = 20) [109]. This suggests neurodegeneration; however, the validity of NAA as a marker has been called into question, as it may include oligodendrocytes and myelin as well as neurons [110].

Changes in the somatosensory cortex may also be functional. A pilot fMRI study demonstrated that people with concurrent painful and insensate DSPN (n = 8) had significantly different blood oxygenation level-dependent signal (BOLD) responses to heat stimulation in the lower limb than people with only painful DSPN (n = 9), painless DSPN (n = 9), no DSPN (n = 9) and healthy controls (n = 9). Functional rearrangement of the primary somatosensory cortex was also observed in this group. The area corresponding to pain in the lower limb expanded to encompass areas of the face and lips, and this response was associated with the severity of DSPN. Multimodal MRI also allowed for the visualization of cortical thickness, with those suffering from painful and insensate DSPN having the greatest reduction in primary somatosensory cortical thickness. Further large-scale studies including people with T2DM are required [111].

### 4.5. Motor and Somatosensory Tracts

A recent large-scale study has been conducted in people with T2DM (painful DSPN n = 23, painless DSPN n = 44) and healthy controls (n = 88), assessing differences in motor and somatosensory tracts using tract-based spatial statistics. When compared to healthy controls, people with DSPN had significantly reduced spatial parameters in white matter tracts, such as the corticospinal, spinothalamic and thalamocortical tracts. In addition, there were significant differences in the pre- and post-central gyrus and deep gray matter nuclei (caudate, putamen, medial pallidum, thalamus and ventral nuclear) [112]. According to an expert comment on this study, the participants enrolled in fact had sub-clinical DSPN based on their mean electrophysiology and questionnaire-based neuropathy outcomes. This is noteworthy because it demonstrates that the CNS is targeted early in disease progression. Longitudinal investigations are needed to better understand the natural history of CNS involvement [113].

## 5. Current Therapies and Future Recommendations

### 5.1. Glycemic Control

Given the importance of chronic hyperglycemia in the pathogenesis of DSPN, glycemic control is currently the most effective disease-modifying therapy and is recommended as a first-line treatment. Its efficacy was most notably demonstrated in the Diabetes Control and Complications Trial/Epidemiology of Diabetes Interventions and Complications (DCCT/EDIC) studies, which found that intensive glucose monitoring therapy reduced the incidence of DSPN in people with T1DM by 69% at 5 years [114]. Its success, however, is limited to this patient group. In people with T2DM, although glycemic control is greatly beneficial, it does not significantly reduce the risk of DSPN. For example, in the UK Prospective Study (UKPDS), a similar trial investigating glucose monitoring but in a T2DM cohort, the rate of DSPN was not significantly reduced (*p* = 0.033) [115]. A Cochrane meta-analysis of 17 RCTs explored these differences and confirmed that glycemic control significantly reduced the risk of DSPN in people with T1DM (annualized risk difference −1.84%) and non-significantly reduced the risk in people with T2DM (annualized risk difference −0.58%) [116]. This may be because people with T2DM have an increased number of risk factors, despite the fact that glycemic medications can also target hyperlipidemia and impair insulin signaling pathways [117]. Interestingly, a study investigating an intervention that targeted glucose, cardiovascular factors, lifestyle and behavior significantly reduced the risk of autonomic neuropathies (hazard ratio 0.37) but not DSPN (hazard ratio 1.09) at 8 years follow-up [118]. Nevertheless, to meet the needs of people with T2DM and DSPN, more emphasis should be placed on the development of therapies that actively target multiple risk factors.

### 5.2. Pain Management

Pain management is a sub-optimal strategy for people with painful DSPN and does not treat the approximately 70% of people with DSPN who do not experience pain [1,119]. Pharmacotherapies include anticonvulsants, serotonin and norepinephrine reuptake inhibitors (SNRIs), tricyclic antidepressants (TCAs), opioids and topical analgesics. Both anticonvulsants and SNRIs have a moderate-quality level of evidence for their effectiveness in reducing pain in DSPN [120,121,122]. However, they are associated with a range of side effects such as drowsiness, dizziness, headache, diarrhea and nausea [3,123,124,125]. Anticonvulsants, in particular, are linked to tachyphylaxis, and there have recently been additional safety concerns raised about an increased risk of severe respiratory depression for people over the age of 65, with respiratory or neurological disease or renal impairment following treatment with pregabalin [126]. The level of evidence for TCAs and opioids is low [127,128], and opioids pose a serious risk with misuse and abuse [129]. Because of this, new guidance advises against the use of opioids in the treatment of DSPN [1]. The level of evidence for topical analgesics in the treatment of localized pain areas in DSPN is moderate to low, but recent studies of 8% topical capsaicin have shown promise in its efficacy [130]. There are concerns, however, that topical capsaicin can cause small nerve fiber injury and, as a result, disturb nociceptive signaling [131]. Pain in DSPN is largely associated with small fiber changes and microvascular dysfunction. Preliminary evidence also suggests that people with painful DSPN have changes in their CNS; however, whether these are upstream or downstream mechanisms is unclear [34]. Further research into the pathogenesis of pain in DSPN is urgently needed. Longitudinal studies, for example, can shed light on the temporal order of nerve fiber, microvasculature and CNS changes, revealing which mechanism is perhaps the most upstream and can be targeted therapeutically [113].

### 5.3. Spinal Cord Stimulation

Based upon the evidence for CNS changes in DSPN, spinal cord stimulation as a neuromodulation therapy for severe painful DSPN has been developed. Similar to pharmacotherapies, the benefit this therapy offers to people with painless DSPN may be limited. The level of evidence for spinal cord stimulation in painful DSPN is low. A recent systematic review found only two multi-center RCTs on spinal cord stimulation for painful DSPN [132]. Although both RCTs were significant in terms of pain reduction [133,134], their designs were of low quality and likely to be biased [132]. A recent Cochrane systematic review (15 RCTs, 908 participants) of the efficacy of spinal cord stimulation in people with chronic pain (not exclusively DSPN) discovered low-quality evidence, as well as the disappearance of any treatment effect once the trials were sham-controlled. Furthermore, it emphasized the serious side effects associated with this therapy, such as infection, lead failure/displacement and a need for further surgical procedures [135]. In addition to continuing to investigate spinal cord stimulation, future research should continue to explore neuromodulation of the dorsal root ganglion, as animal studies alternatively suggest this area as the origin of axonal loss [46,47]. At present, there have been case series exploring this novel treatment [136,137], but no RCTs have been published investigating its efficacy.

## 6. Conclusions

DSPN is a progressive and debilitating complication of diabetes, with current management strategies failing to achieve long-term efficacy. Glycemic control is an effective therapy for people with T1DM but fails to significantly reduce the risk of DSPN in people with T2DM. Pain management may treat painful DSPN (but not other symptoms), and these pharmacotherapies are linked to a variety of side effects. Spinal cord stimulation is a novel neuromodulation therapy that acts upon the CNS; however, the current level of evidence for its use in severe painful DSPN is low, and it is linked to serious procedural risks. Researchers must focus their efforts on developing novel disease-modifying therapies; however, further understanding of the pathogenesis is required in order to develop beneficial therapies with a strong preclinical evidence base. Animal and cell models, as well as human nerve samples from people with DSPN, have revealed that the neuropathological features include a breakdown in the myelin sheath, dysfunctional Schwann cells, disrupted axonal transport, microvascular disease and eventually neuronal loss. Hyperglycemia is the most studied metabolic mechanism and contributes to the development of DSPN by activating the polyol, glycation, PKC, PARP and hexosamine pathways, all of which cause oxidative stress and damage nerves and microvessels. Hyperlipidemia is also serious and can be particularly problematic for people with T2DM, whereby the oxidation of excess fatty acids promotes pathogenic mechanisms. Impaired insulin signaling affects people with T1DM and T2DM and may reduce neuronal survival via different mechanisms. Advances in neuroimaging studies have shown that DSPN is associated with structural and functional changes in the CNS as well as in the peripheral nervous system. These findings are mostly limited to pilot studies, but highlight the need for more research in this area, where data are scarce, and many aspects remain unexplored. In addition, the definition of DSPN should be revised to consider CNS changes, and the common term “diabetic peripheral neuropathy” should be abandoned. These recent insights have altered the disease model and raise the prospect of new biomarkers and therapeutic targets. Priority should be given to the discovery of any upstream linked mechanisms that a between the peripheral and central nervous systems and the microvasculature, and interventions that may target multiple risk factors.

## Data Availability

Not applicable.
